# Clinical Study of 8 Cases of *CHD2* Gene Mutation–Related Neurological Diseases and Their Mechanisms

**DOI:** 10.3389/fcell.2022.853127

**Published:** 2022-03-21

**Authors:** Xiaona Luo, Xiaoang Sun, Yilin Wang, Longlong Lin, Fang Yuan, Simei Wang, Wenjing Zhang, Xiaobing Ji, Meiyan Liu, Shengnan Wu, Xiaoping Lan, Jie Zhang, Jingbin Yan, Fanyi Zeng, Yucai Chen

**Affiliations:** ^1^ Department of Neurology, Shanghai Children’s Hospital, Shanghai JiaoTong University, Shanghai, China; ^2^ Department of Clinical Laboratory, Shanghai Children’s Hospital, Shanghai Jiao Tong University, Shanghai, China; ^3^ NHC Key Laboratory of Medical Embryogenesis and Developmental Molecular Biology and Shanghai Key Laboratory of Embryo and Reproduction Engineering, Shanghai, China

**Keywords:** chromodomain helicase DNA-binding protein 2 *(CHD2)* gene, neurological diseases, epilepsy, repressor element 1-silencing transcription factor, expression

## Abstract

**Background:** The chromodomain helicase DNA-binding protein 2 (*CHD2*) gene, is an ATPase and part of the *CHD* family of chromatin remodelers. Mutations in the *CHD2* gene are inherited in an autosomal-dominant manner and can lead to intellectual disability, epilepsy, and autism. We investigated the clinical characteristics of *CHD2*-related conditions and their possible pathogenesis.

**Methods:** We collected and analysed the clinical data of patients that were identified as having *CHD2* mutations. Genetic testing was performed using targeted sequencing or whole-exome sequencing. We analysed the expression of *CHD2* and repressor element 1-silencing transcription factor (*REST*) in blood samples using quantitative PCR and the conservation of the mutations. The *CHD2* mutations we identified were compared with the known mutations reported in the *CHD2*-related literature.

**Results:** Eight patients with *CHD2* gene mutations were analysed. Six mutations were identified; four were unreported previously (c.670C>T; c.4012A>C; c.2416dup; c.1727–1728insAT), and two were known mutations: c.5035C>T (two cases) and c.4173dup (two cases). Among these mutations, seven were *de novo* mutations, and one could not be determined because the parents refused genetic testing. The clinical manifestations included mild or severe intellectual disability, epilepsy, and behavioural abnormalities. Quantitative PCR showed that the *CHD2* gene expression levels among the patients, parents, and the controls were not significantly different. The levels of *REST* gene expression in the patients were significantly higher than those of the controls; thus, mutation of the *CHD2* gene led to an increase in the expression level of the *REST* gene. The mutations reported were all located in conserved positions in different species. Among the various medications administered for treatment, valproate showed the best results for the treatment of epilepsy caused by *CHD2* gene mutation.

**Conclusion:** Mutation in *CHD2* did not lead to a significant decrease in its expression level, indicating that the clinical phenotype was unrelated to its expression level, and the mutant protein may retain some function. Most of the mutations relatively stable. In addition, the clinical manifestations from the same mutation in the *CHD2* gene were different among the known cases; this may be related to the regulation of *REST* or other regulatory factors.

## Introduction

The chromodomain helicase DNA-binding protein 2 (*CHD2*) is a member of the ATP-dependent chromatin remodelling family of proteins that are critical for the assembly and regulation of chromatin (Lamar and Carvill, 2018). The *CHD2* gene is located on the long arm of chromosome 15 and encodes a member of the *CHD* family of proteins (Wilson et al., 2021). The *CHD2* gene contains 42 exons that encode 1828 amino acids. The *CHD2* protein is composed of several functional domains, including two chromodomains at the N-terminus, an ATPase/helicase domain, and a DNA-binding domain. The N-terminal region of *CHD2*, which contains tandem chromodomains, serves an auto inhibitory role in both the DNA-binding and ATPase activities of *CHD2* (Liu et al., 2015).

In 2009, a microdeletion of 15q26.1 was identified in a 30-month-old female who had refractory myoclonic epilepsy with intellectual disability, growth retardation, and a distinctive facial appearance (Veredice et al., 2009). In 2013, the gene primarily responsible for the epileptic component, and the disruption of neural developmental directly caused by seizures that resulted from the 15q26 defect, was *CHD2* (Suls et al., 2013; Capelli et al., 2012; Epi4K Consortium et al., 2013; Poot et al., 2013). Since then, several mutations in the *CHD2* gene have been identified and associated with developmental delay, microcephaly, autism, and epilepsy (Epi4K Consortium et al., 2013; Poot et al., 2013; Carvill et al., 2013; O'Roak et al., 2014; Deciphering Developmental Disorders Study, 2017). Seizures typically develop as early as 6 months and before 4 years of age; the multiple seizure types include myoclonus, myoclonic-absence seizures, and drop attacks (Capelli et al., 2012; Lamar and Carvill, 2018; Nieto-Estevez and Hsieh, 2018; Chen et al., 2020).

This study reports the cases of eight patients with *CHD2* gene mutations and describes their clinical characteristics, laboratory examinations, electroencephalogram (EEG) results, mutational genotypes, and mRNA expression levels of *CHD2* and repressor element 1-silencing transcription factor (REST). To our knowledge, all CHD2 mutations reported so far are heterozygous and the vast majority are new mutations. At present, the mechanism of CHD2 mutation to autosomal dominance is not clear. In this paper, we will preliminarily study and speculate the cause. Besides we found that the expression of REST gene was significantly increased in patients with CHD2 mutation, but the specific mechanism is still unclear and needs further study.

## Materials and Methods

### Patients

Venous blood samples from eight patients suspected of having *CHD2* mutations were collected in the Shanghai Children’s Hospital from September 2019 to June 2021 after informed consent was obtained from the patients and/or their families. Data about age at seizure onset, seizure types, birth history, family history, EEG and brain magnetic resonance imaging (MRI) results, and treatment were also collected. This study was approved by the Ethics Committee of Shanghai Children’s Hospital, and all protocols complied with Chinese bioethics laws and the Declaration of Helsinki.

### Genetic Analysis and cDNA Synthesis

The screening for mutations in *CHD2* (RefSeq NM_001271.3) was performed using epilepsy-targeted next-generation sequencing (P6) or whole-exome sequencing (WES, P1-P5 and P7-P8). This study was approved by the Ethics Committee of Shanghai Children’s Hospital (approval No. 2019R071-F03). Variants calling and interpretation have been described elsewhere (Xiaozhen et al., 2021).

Total RNA was extracted from 0.25 ml of fresh blood using an RNA extraction kit (YEASEN Biotech Co., Ltd., Shanghai, China) according to the manufacturer’s instructions (Qiagen). The concentration and purity of the total RNA were detected using a nucleic acid–protein analyser. Next, cDNA was synthesized using the total RNA as the template for reverse transcription according to the manufacturer’s instructions of the reverse transcription kit. The reaction contents were 5 μl template RNA (≤500 ng), 2 μl Primer Script RT Enzyme Mix, and 3 μl RNase-free water. The samples were incubated at 37°C for 15 min and then 85°C for 5 s.

### Quantitative PCR Analysis of *CHD2* and *REST* Gene Expression

Because two families refused a second collection of blood samples, cDNA made from the blood of the core members of the remaining six families and control peers was used for fluorescence quantitative PCR (qPCR), which was carried out using a real-time fluorescence qPCR kit (Takara Biomedical Technology) according to the manufacturer’s instructions. Due to an insufficient blood sample in Case 5, the *REST* gene expression level could not be determined. The primers used to measure the expression of *CHD2* were (forward) 5′-CGA​AAA​CAG​GCA​CTG​GAC​CAC​T-3′ and (reverse) 5′-GAT​GAC​GAC​TGT​GTC​CGC​TGA​A-3′. The *REST* primers were (forward) 5′-CCC​CTT​CAC​ATG​GAG​CCA​AT-3′ and (reverse) 5′-CTT​CCG​TGC​CCT​TTC​ACT​CT-3′. The reaction (20 μl) contained cDNA (2 μl), forward primer (10 μM; 0.4 μl), reverse primer (10 μM; 0.4 μl), 2× SYBR Premix Ex Taq (10 μl), and nanopure water (7.2 μl). Housekeeping gene GAPDH was used as a relative quantity. Statistical analysis. Data are presented as means ± s.e.m. from three independent experiments. Each experiment was performed in duplicate. Data were analyzed using the Student’s t-test, and *p* < 0.05 was considered to be statistically significant.

### Conserved Sequence Analysis

We analyzed the mutations in the *CHD2* protein sequence for H.Sapiens, M.Mulatta, C.Lupus, M.Muschulus, R.Norvegicus, G.Gallus, D.Rerio and D.Melanogaster to predict sequence conservation at these loci.

## Results

### Clinical Data

**FIGURE 1:**
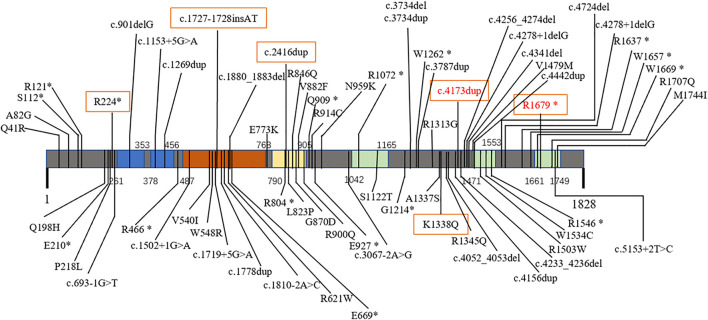
Mutations of *CHD2*-related epilepsy in different domains. Primary structure and mutations in the *CHD2* protein: chromodomain 1 (261–353) and 2 (378–456) (blue); SNF2-related helicase domain (487–768, brown) with DEAD-like helicase superfamily (503–648) and helicase ATP-binding (496–666) domains; ATP helicase domain (790–905, yellow); and domains of unknown function (1042–1165, 1471–1553, 1661–1749, green). Red text: previously reported mutations. *Boxed text*, mutations in this report.

The clinical characteristics of the patients, the *CHD2* gene mutations identified in the patients, and their treatments are presented in Table 1. In all, eight children with *CHD2* gene mutations in seven families were analysed. These included three males and five females, with six mutations, including two mutations that had been reported previously (c.5035C>T, p. R 1679*; and c.4173dup, p. Q 1392 T*17), and four mutations had not been reported (c.670C> T, p. R 224*; c4012A>C, p. K 1338Q; c.2416dup, p. R806Kfs*20; and c.1727–1728insAT, p. E576Efs*13) (Figure 1). All of these mutations were *de novo* in the children; in Case 6, the parents refused genetic examination.

**TABLE 1:** Clinical data in this study.

Age of seizure onset	Sex	Clinical manifestation	Gene mutation	Zygotic state	Gene sequencing method	Inheritance	Reported	Head MRI	Treatment
4 years	F	GTCS, partial tonic, partial tonic–clonic	c.5035C>T	Het	WES	*De novo*	Yes	Normal	VPA
			R1679*						
4 years	F	GTCS (10–15 min), partial tonic	c.5035C>T	Het	WES	*De novo*	Yes	Normal	VPA
			R1679*						
40 days	M	GTCS, eyelid myoclonic, infantile spasm	c.4173dup	Het	WES	*De novo*	Yes	Less cerebral white matter	PB → VPA, TPM, LTG, ACTH, KD
			Q1392T*17						
	F	Abnormal EEG, hyperactivity, mild intellectual disability	c.4173dup	Het	WES	*De novo*	Yes	Noncompliance	None
			Q1392T*17						
5 years	M	GTCS (10 min, grumpy, talking to himself	c.670C>T	Het	WES	*De novo*	No	Abnormal (concrete is unknown)	VPA
			R224*						
5 years	M	Partial tonic–clonic, only disturbances of consciousness, frequent seizures	c.4012A>C	Het	epilepsy-targeted next-generation sequencing	Unknown	No	Normal	LEV, VPA, CBZ
			K1338Q						
3 years, 5 months	F	GTCS, myoclonic, partial tonic, partial tonic–clonic (20 min), frequent seizures	c.2416dup	Het	WES	*De novo*	No	Normal	LEV, VPA, TPM, CLZ
			R806Kfs*20						
3 years, 1 months	F	GTCS, partial tonic–clonic (20 min), frequent seizures	c.1727-1728insAT	Het	WES	*De novo*	No	Small hippocampus	LEV, VPA, TPM, PER, KD
			E576Efs*13						

GTCS, generalized tonic–clonic seizure; VPA, valproate; PB, phenobarbital; TPM, topiramate; LTG, lamotrigine; CBZ, carbamazepine; CLZ, clonazepam; PER, perampanel; ACTH, adrenocorticotropic hormone; KD, ketogenic diet; Het, heterozygous.

Each of the eight patients had neurological abnormalities; seven had seizures, and the other one had growth retardation with multiple abnormal EEG discharges. The initial seizure age ranged from 40 days to 5 years. The types of epilepsy were generalized tonic–clonic seizure, eyelid myoclonic, myoclonic, tonic, partial tonic, partial tonic–clonic, consciousness-only disturbances, and infantile spasm. Five patients had status epilepticus, and four patients had frequent onset. Case 1 and Case 2 were identical twin sisters; the other children were unrelated. In Case 3, a caesarean section was performed before birth due to insufficient foetal movement. The child had a history of hypoglycaemia 3 days after birth, and spasticity occurred 40 days after birth. Case 6 had polydactyl deformity. The other children had no abnormal birth history. The EEG results were consistent with the clinical patterns.

Case 1 and Case 2 were twin sisters who were delivered by caesarean section at 36 weeks of gestation. Oxygen was administered through a nasal catheter after birth; the rest of the birth history was normal. They carried a *de novo* c.5035C>T (p.R 1679*) *CHD2* mutation, which has been reported previously (Wang et al., 2017).Originally they had status epilepticus, and EEG suggests epileptic discharge, and photosensitivity. The two patients had mild developmental delays before onset and an IQ of 70; after controlling the epilepsy using valproate (VPA), an EEG examination did not find abnormalities.

Case 3 was a male with no abnormal birth history, an age of seizure onset of 40 days, who carried a *de novo* c.4173dup (p. Q 1392T*17) *CHD2* mutation, which has been reported previously (Chen et al., 2020). He was admitted to the neonatal intensive care unit 3 days after birth due to hypoglycaemia and developed seizures 40 days after birth with clinical manifestations of eyelid myoclonus, tonic–clonus, tonics, and infantile spasms. An EEG showed hypsarrhythmia. For treatment, phenobarbital → VPA + topiramate (TPM) + adrenocorticotropic hormone (ACTH) + lamotrigine (LTG) → pulse methylprednisolone + ACTH + ketogenic diet (KD) were successively administered. After the seizure was controlled, the EEG was improved, but there was still developmental delay; she could not sit up without assistance at 11 months of age, and a head MRI exam suggested less white matter.

Case 4 was a female with no abnormal birth history who was admitted to our department due to hyperactivity and intellectual disability. Her IQ was 79. Her EEG was abnormal: epileptic discharge in the Rolandic area during sleep; previously, the head predominated with slightly more epileptic discharge, and the slow complex wave index in non-rapid eye movement sleep was about 60–65%. The *CHD2* gene had a *de novo* c.4173dup (p. Q 1392T*17) mutation, which is the same as in Case 3. However, unlike Case 3, the child presented only developmental delay and abnormal behaviour but no seizures.

Case 5 was a male with no abnormal birth history who developed a generalized tonic–clonic seizure at the age of 5 years. An EEG showed that in the two occipital regions, there were several bursts of diffuse irregular spikes/spines/multi-spines that were emitted in short segments. After treatment with VPA, the seizures were controlled. After 4 years of drug withdrawal, one seizure occurred, and there was abnormal mental behaviour. The patient carried a *de novo* c.670C>T (p.R 224*) *CHD2* mutation, and his IQ was 63.

Case 6 was a 5-year-old male who underwent caesarean section due to insufficient foetal movement at birth. He had multiple malformations of the little finger/toe of his right hand and right foot, and his growth and development showed regression. The epilepsy showed disturbances of consciousness and partial ankylosis, with frequent seizures at the beginning of the disease; the seizures continued after LEV+VPA treatment but were controlled with the addition of carbamazepine. The patient carried a c.4012A>C (p. K 1338 Q) *CHD2* mutation; the parental genes could not be analysed due to refused consent.

Case 7 was a 3.5-year-old female with no abnormal birth history. She had frequent seizures at 3.5 years, with general tonic–clonic as the clinical manifestation. Her growth and development before onset were normal for her age, and seizures were frequent and difficult to control at the beginning of the disease. After LEV+VPA+TPM+pirampanet+KD treatment, the seizures were controlled. She carried a *de novo* c.2416dup (p.R806Kfs*20) *CHD2* mutation.

Case 8 was a 3-year-old female with no abnormal birth history. She developed tonic–clonic seizures at the age of 3 years, with frequent seizures and initial difficulty in control. After treatment with LEV+VPA+TPM+CLZ, she had seizure control. After the onset of the seizures, there was developmental regression and mild mental behaviour abnormalities. She carried a *de novo* c.2416dup (p.R806Kfs*20) mutation in *CHD2*.

### qPCR Analysis of *CHD2* and *REST* Gene Expression

**FIGURE 2:**
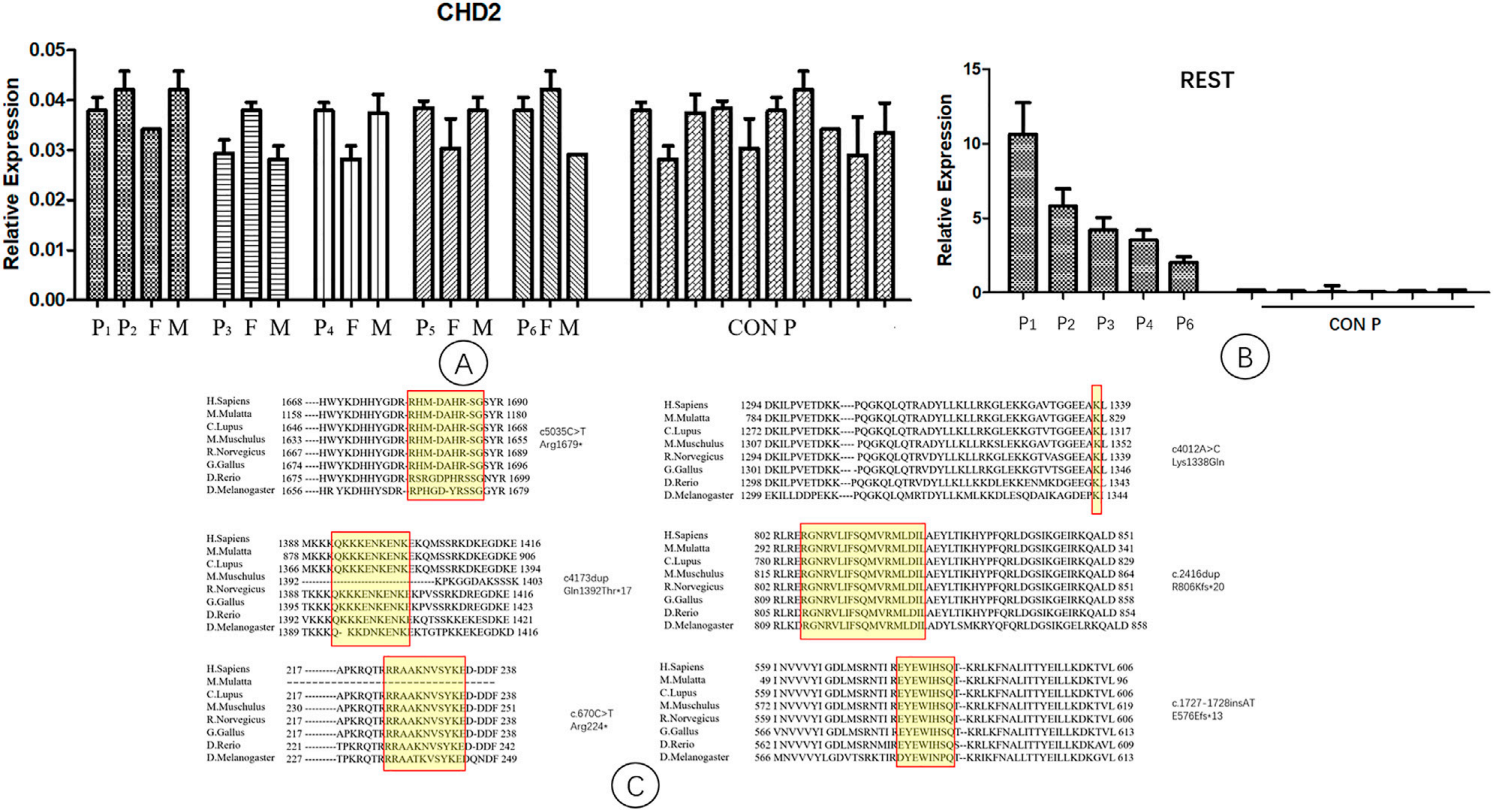
Quantitative PCR analysis of *CHD2* and *REST* gene expression. P1-P6, patient 1–6; F, father; M, mother; CON P, control group. Arabic numerals represent the age of the control group. **(A)** The expression levels of the *CHD2* gene in patients, parents, and the controls. They were not significantly different. **(B)** The expression levels of the *REST* gene in patients and the controls. The levels of *REST* gene expression in the patients were significantly higher than those of the controls. **(C)** Conservation studies were conducted for *CHD2* mutations reported in this manuscript in H.Sapiens, M.Mulatta, C.Lupus, M.Muschulus, R.Norvegicus, G.Gallus, D.Rerio, D.Melanogaster. All the mutations in this study were relatively stable.

The expression levels of *CHD2* in the six families and the controls were not significantly different (Figure 2A). The *REST* gene expression levels in the five patients were significantly higher than those of the controls (Figure 2B).

### Conservation of Mutations

Conservation studies were conducted for all the mutations of this study in H.Sapiens, M.Mulatta, C.Lupus, M.Muschulus, R.Norvegicus, G.Gallus, D.Rerio and D.Melanogaster (Figure 2C). These analyses suggested that the mutations relatively stable.

## Discussion

Mutations in the *CHD2* gene can lead to neurodevelopmental delay, intellectual disability, epilepsy, and behavioural problems. Hanly et al. (Hanly et al., 2021) analysed 56 *CHD2* gene mutations through a literature search and found that 80.3% (45 cases) of the patients had epilepsy; the incidence of epilepsy in our study was 87.5%. While most *CHD2* mutations are *de novo*, a few are inherited from asymptomatic parents. To our knowledge, only Petersen et al. (Petersen et al., 2018) has reported a case of an inherited symptomatic CHD2 mutation, which affected both mother and daughter.

A search of the Human Gene Mutation Database (2020.6) shows that 128 cases of *CHD2* mutation have been reported with 103 mutation sites. Missense mutations are the most common, followed by frameshift and nonsense mutations; splice mutations are uncommon, and other mutations are rare (Table 2). *CHD2* is a relatively conserved gene, and many loci are relatively conserved in multiple species. These missense mutations that have been reported are mostly at these conserved loci. Among the 128 cases reported, the associated *CHD2* mutations included c.693-1G>T (two cases), c.1809+1delG (three cases), c.1942C>T (two cases), c.2567A>G (three cases), c.2895_2898delAGAA (three cases), c.3521G>A (two cases), c.3734delA (two cases), c.3787dupG (two cases), c.4173dupA (four cases), c.4233_4236delAGAA (two cases), c.4799_4814delACCTTCACCCTCAGAA (three cases), c.4909C>T (four cases), c.4949dupG (three cases), c.5035C>T (three cases), and other mutations (one case). There seems to be no difference in the number of mutation sites of *CHD2*-related epilepsy in different domains (Figure 1).

**TABLE 2:** *CHD2* mutations reported before.

Mutation type	Number
Frameshift	23
Inframe	1
Missense	47
Noncoding	2
Nonsense	20
Splice	10
Total	103

Photosensitivity is often present, and self-induced seizures are seen in some patients (Suls et al., 2013; Galizia et al., 2015; Thomas et al., 2015). In this study, Case 1 and Case 2 showed photosensitivity, which is consistent with previous reports. Zebrafish larvae with a *CHD2* knockout exhibit epileptic behaviour (Suls et al., 2013); *CHD2* morpholino-injected larvae display body curvature, excessive body pigmentation, and a developmental delay (Suls et al., 2013). *CHD2* ^+/−^ mice showed an increase in pyramidal neuron action potential discharge, a decrease in spinous process adaptation, significantly increased amplitude of micro-excitatory postsynaptic currents, and a significantly decreased frequency of micro-inhibitory postsynaptic currents. EEG recordings from the somatosensory neocortex of *CHD2* ^+/−^ mice show increased frequency ranges of α, δ, θ, and γ waves, suggesting that *CHD2* affects synaptic transmission of glutamate and γ-aminobutyric acid (GABA) and cortical rhythmgenesis in the adult hippocampus (Kim et al., 2018; Nieto-Estevez and Hsieh, 2018). This suggests that *CHD2* mutation leads to a prominent reduction of GABA and an increase in neuronal excitability that results in seizures; this could be verified by further studies.

Two cases of the previously reported c5035C>T mutation were found in identical twin sisters (Cases 1 and 2). Their age of epilepsy onset was 4 years, and their clinical manifestations included complex partial seizures that initially presented as a persistent epileptic state and an EEG that suggested photosensitivity; these are consistent with previous reports (Wang et al., 2017).

Two cases of the p. Q 1392T*17 mutation were reported in this study, and five cases have been previously reported at this site (Lund et al., 2014; Galizia et al., 2015; Pinto et al., 2016; Chen et al., 2020), all of which were *de novo* and presented mainly as Lennox–Gastaut syndrome and autistic disorder. The reported manifestations included eyelid myoclonus, myoclonus, atonia, and atypical absence; all of the patients carrying this mutation had intellectual disability. However, the two cases we report in the present study had different clinical manifestations. One presented infantile spasm, while the other presented only mild intellectual disability and behavioural abnormalities, an abnormal EEG, and no seizures; these indicate that there are different clinical manifestations of the same gene mutation at the same site. Children with epilepsy inherited from asymptomatic fathers have been reported in the past (Chen et al., 2020). A *CHD2* mutation that affected both mother and daughter has been reported with varying clinical manifestations (Petersen et al., 2018). This further supports the lack of a genotype–phenotype correlation among individuals with the same genetic variant, and suggests that *CHD2* gene effects might be mediated by other mechanisms. It has been demonstrated that *CHD2* mRNA is regulated in some tissues by the ubiquitous splicing regulator Rbfox2 (Cho et al., 2019), and *CHD2* probably binds directly to *REST* genomic DNA to regulate the expression of *REST* (Shen et al., 2015). This suggests that different clinical manifestations may be related to these regulatory factors.

Mice with homozygous deletions of the C-terminus of CHD2 exhibit perinatal lethality, and heterozygous mice exhibit decreased survival rates, suggesting that *CHD2* is an essential gene for normal development (Marfella et al., 2006). All the patients in this study had heterozygous mutations, and there were no homozygous or complex heterozygous mutations reported in the past, suggesting that the *CHD2* gene is essential for human growth and development. Suls et al. (Suls et al., 2013) performed qPCR on two cases of nonsense mutations and found that the expression level was not decreased, which is consistent with the present study and suggests that the aberrant allele is not degraded by nonsense-mediated mRNA decay. Analysis using qPCR performed on a patient with a splicing mutation showed that both alleles were expressed (Suls et al., 2013). It is suggested that the *CHD2* mutation does not affect the RNA expression level, and that the protein translated from the mutated *CHD2* gene has some functionality; this mutant protein might affect the excitability of GABAergic neurons or other electrophysiological channels, resulting in increased neuronal excitability and leading to seizures.

Recent studies have shown that *CHD2* is expressed throughout brain tissue, but varies in different cell subtypes at different developmental stages (Shen et al., 2015; Wilson et al., 2021). In mature mice (30 days after birth), *CHD2* is significantly expressed in the olfactory bulb, neocortex, hippocampus, and cerebellum (Kim et al., 2018). In *CHD2* ^+/−^ mice, there are fewer GABAergic interneurons in the somatosensory cortex, and the density of GABA neurons in the cortex is decreased; the loss of GABA intermediate neurons affects hippocampal memory (Kim et al., 2018). *CHD2* is critical for embryonic and adult neuronal differentiation, synaptic properties, and memory; this partially explains the symptoms of *CHD2* patients (Kim et al., 2018). Brain MRI of Case 8 in this study suggested that a reduction of the hippocampal volume might be related to the *CHD2* mutation. During embryonic development, there is a decrease in proliferative cells in the ventricular zone and subventricular zone as well as in NKX2.1+ progenitor cells in the medial and caudal ganglionic eminence (Kim et al., 2018). CHD2 is highly expressed in the Pax6+ radial glia but rarely expressed in Tbr2+ intermediate progenitors, suggesting that CHD2 deficiency suppresses the self-renewal capacity of the radial glia and instead promotes premature neuronal differentiation (Shen et al., 2015; Lamar and Carvill, 2018). This early differentiation probably leads to rapid depletion of the progenitor pool, resulting in a smaller cortex and defects in later-born neurons (Homem et al., 2015; Lamar and Carvill, 2018). In human cortical interneurons derived from human embryonic stem cell cultures, Meganathan and others (Shen et al., 2015) found that CHD2 expression increases during cortical interneuron differentiation.

Knockout of the *CHD2* gene in human embryonic stem cell–derived interneurons results in a decrease in the number of neurons and a reduction in the neurite length (Meganathan et al., 2017). Among the eight patients in this report, head MRI indicated widening of the cerebral sulcus in four cases; in one case head MRI imaging data could not be obtained; cranial MRI did not show abnormalities in the other three cases. Except for two patients that had normal development before the onset of epilepsy, the rest of the patients had different degrees of slowed mental development; the two patients experienced mental regression after the seizure onset.

*CHD2* probably binds directly to the *REST* genomic DNA to regulate the expression of *REST*; the *in vitro* overexpression of *REST* rescues the defect in neurogenesis caused by *CHD2* knockdown (Shen et al., 2015). To evaluate the relationship between *CHD2* and *REST in vivo*, we measured the expression levels of *REST* mRNA in patients with *CHD2* mutations. We found that the expression level of *REST* mRNA in the peripheral blood of patients with *CHD2* mutations was significantly higher than that of the control (wild type) group. This suggests that the mutation of the *CHD2* gene could result in high expression of *REST*, which might alleviate the clinical symptoms caused by the *CHD2* mutation and cause the lack of a genotype–phenotype correlation mentioned above.

A total of six *CHD2* mutation sites were reported among eight cases; of these, four mutation sites were previously unreported; this identifies new sites for pathogenic *CHD2* mutations. The c.4173dupA mutation was reported in five previous cases, and two more cases are reported in this paper, suggesting that this site may be a hotspot for mutation. The pathogenesis of epilepsy caused by *CHD2* is still unclear and may be related to decreased GABA activity. In this study, seven patients with epilepsy were treated with VPA to control their seizures, and it has been reported that VPA has good effect in the treatment of *CHD2* mutation (Chen et al., 2020), but for patients with refractory epilepsy, a combination of medications or other methods are needed to control epilepsy. At present, the pathogenesis caused by *CHD2* gene mutations is not clear, and further research is needed to elucidate the mechanism. Two possible causes of the phenotypic effects of a *CHD2* gene mutation are as follows: *1*) the mutant protein might still have some function and can lead to the dysfunction of another normal protein, and *2*) the gene mutation results in reduced GABAergic channel activity, which can lead to seizures. These speculations need to be tested by further study.

## Data Availability

Publicly available datasets were analyzed in this study. This data can be found here: http://archive.uwcm.ac.uk/uwcm/mg/hgmd0.html.
